# CrysFormer: Protein structure determination via Patterson maps, deep learning, and partial structure attention

**DOI:** 10.1063/4.0000252

**Published:** 2024-08-14

**Authors:** Tom Pan, Chen Dun, Shikai Jin, Mitchell D. Miller, Anastasios Kyrillidis, George N. Phillips

**Affiliations:** 1 Department of Computer Science, Rice University, Houston, Texas 77005, USA; 2 Department of BioSciences, Rice University, Houston, Texas 77005, USA; 3 Department of Chemistry, Rice University, Houston, Texas 77005, USA

## Abstract

Determining the atomic-level structure of a protein has been a decades-long challenge. However, recent advances in transformers and related neural network architectures have enabled researchers to significantly improve solutions to this problem. These methods use large datasets of sequence information and corresponding known protein template structures, if available. Yet, such methods only focus on sequence information. Other available prior knowledge could also be utilized, such as constructs derived from x-ray crystallography experiments and the known structures of the most common conformations of amino acid residues, which we refer to as partial structures. To the best of our knowledge, we propose the first transformer-based model that directly utilizes experimental protein crystallographic data and partial structure information to calculate electron density maps of proteins. In particular, we use Patterson maps, which can be directly obtained from x-ray crystallography experimental data, thus bypassing the well-known crystallographic phase problem. We demonstrate that our method, CrysFormer, achieves precise predictions on two synthetic datasets of peptide fragments in crystalline forms, one with two residues per unit cell and the other with fifteen. These predictions can then be used to generate accurate atomic models using established crystallographic refinement programs.

## I. INTRODUCTION

Proteins, the molecular machines of living systems, play a central role in most cellular processes.^1^ Investigating a protein's structure is a classic challenge in biology, given that its function is dictated by its specific conformation. Proteins comprise long chains of linked, relatively small organic molecules called *amino acids*, with twenty considered standard. However, these underlying polypeptide chains fold into complex three-dimensional structures, as well as into larger assemblies thereof. Consequently, biologists aim to establish a standardized approach for experimentally determining and visualizing the overall structure of a protein at a low cost.

In the past decades, there have been three general approaches to the protein structure problem: (i) ones that rely on physical experimental measurements, such as x-ray crystallography, NMR, or cryo-electron microscopy; see Ref. 2 for more details; (ii) protein folding simulation tools based on thermodynamic or kinetic simulation of protein physics;^3,4^ and (iii) evolutionary programs based on bioinformatics analysis of the evolutionary history of proteins and their amino acid sequences.^5,6^

Recent advances in machine learning (ML) algorithms have inspired a fourth direction, which is to train a deep neural network model on a combination of a large-scale protein structure dataset (i.e., the Protein Data Bank^7^) and knowledge of the amino acid sequences of a vast number of homologous proteins, to directly predict the protein structure from the protein's amino acid sequence. Recent research projects—such as AlphaFold2^8^—further show that, with co-evolutionary bioinformatic information (e.g., multiple sequence alignments), deep learning can achieve highly accurate predictions in many cases.

The experimental method remains the authoritative source, often providing needed accuracy in atomic locations and further knowledge about interactions of the proteins with other entities such as metals, small molecules, and other proteins.

### A. Our hypothesis and contributions

While it is true that computational methods of predicting structures without experimentally confirming data are improving, they are not yet complete—in terms of the types of structures that can be predicted—and suffer from lack of accuracy in many of the details.^9^ X-ray crystallographic data continue to be a gold standard for critical details describing chemical interactions of proteins.

A robust and accurate way of going directly from an x-ray diffraction pattern of a protein crystal to a solved structure would be a solid contribution to x-ray crystallography. However, both recent initial work^10^ (based on residual convolutional autoencoders) on applying machine learning to this task, as well as the work we present here, has only been applied to relatively small molecules whose crystal structures have a high percentage of solvent in their unit cells. This condition is rare for actual proteins,^11–13^ and such structures can very often be solved with existing *ab initio* direct methods such as SHELXD.^14^ Still, our work is a key intermediate step on the path toward a general ML-based approach for interpreting and deriving structures from crystallographic diffraction patterns.

Here, we present a hybrid convolution and transformer-based model, named CrysFormer, that utilizes protein crystallography information in the form of Patterson maps to directly predict the electron density maps of proteins. To the best of our knowledge, along with Ref. 10, these are the first works to attempt this setting. Crystallographic refinement procedures such as *PHENIX Autobuild* can be applied to electron density maps to obtain protein structures in the form of atomic coordinates. CrysFormer can incorporate “partial structure” information when available; such information could be included in existing solutions that neglected this feature, like the convolutional U-Net-based architectures in Ref. 10. While we are still not yet ready to solve real problems, we successfully solve a simplified problem. As a highlight, using a new dataset of small peptide fragments of variable unit cell sizes—a by-product of this work—we demonstrate that our method can achieve more accurate predictions than the state-of-the-art^10^ with fewer computations.

## II. PROBLEM SETUP AND RELATED WORK

### A. X-ray crystallography and the crystallographic phase problem

X-ray crystallography has been the most commonly used method to determine a protein's three-dimensional structure for over 100 years.^15^ The end result of an application of this method is an electron density map,^16^ to which refinement programs can be applied to obtain atomic structures. However, an open question, the crystallographic phase problem, prevents researchers from directly using raw experimental measurements to obtain accurate structures/electron density maps.

In review, each measured spot (known as a reflection) in an x-ray crystallography diffraction pattern is denoted by three indices *h*, *k*, and *l.*^17^ Any reflection has an underlying mathematical representation, a structure factor, dependent on the locations and scattering factors of all the atoms within the crystal's unit cell

$$F(h,k,l)=\sum_{j=1}^{n}f_{j}\cdot e^{2\pi i(hx_{j}+ky_{j}+lz_{j})},$$ (1)

where the scattering factor and location of atom *j* are *f_j_* and $\left(x_{j},y_{j},z_{j}\right)$, respectively.

A structure factor F(h,k,l) is a complex number and thus has both an amplitude and a phase component (denoted by ϕ). Then, to produce an accurate estimate of the electron density at any point (*x*, *y*, *z*) within the crystal's unit cell, we can take a complex Fourier transform of all of these structure factors, as in

$$\rho (x,y,z)=\frac{1}{V}\cdot \sum_{h,k,l}|F(h,k,l)|\cdot e^{-2\pi i(hx+ky+lz-\phi (h,k,l))},$$ (2)

where *V* is the volume of the unit cell. The amplitude |F(h,k,l)| of any structure factor is easy to determine, as it is simply proportional to the square root of the measured intensity of x rays from the corresponding reflection. However, it is impossible to directly determine the phase ϕ(h,k,l) of a structure factor, well-known as the crystallographic phase problem.^15^

### B. Solving the phase problem

Various methods have been developed to solve the crystallography phase problem for proteins. The three commonly used methods are isomorphous replacement, anomalous scattering, and molecular replacement.^15,18^ Molecular replacement (MR) in particular depends on the availability of an existing structure similar to the desired one. Following the introduction of AlphaFold2,^8^ predictions thereof have been shown to be effective when used as search models for this technique.^19^ This concept was further developed into an iterative procedure involving multiple rounds of MR, crystallographic refinement, and model building.^20^ Also, what is known as direct methods have been successful for small molecules, or in some cases proteins that diffract to atomic resolution. Still, they rarely work for protein crystallography due to the difficulty of resolving atoms as separate objects.

Alternative methods have been developed to solve the phase problem based on intensity measurements alone, known as phase retrieval.^21–23^ However, these methods have not been widely used in x-ray crystallography because they assume different sampling conditions or were designed for non-crystallographic fields of physics. The iterative non-convex Gerchberg–Saxton algorithm^24,25^ is a well-known example of such methods but requires more measurements than are available in crystallography. Although adaptations of the Gerchberg–Saxton algorithm have been proposed for crystallography-like settings, they have not been used to solve the phase problem except in exceptional cases where crystals have very high solvent content.^11–13^ More recently, Candes *et al.*^26^ introduced the Phaselift method, a convex, complex semidefinite programming approach, while Candes *et al.*^27^ introduced the Wirtinger flow algorithm,^27^ a non-convex phase retrieval method. Both these methods have not been applied practically, due to their computationally intensive nature.

## III. CrysFormer: USING 3D MAPS AND PARTIAL STRUCTURE ATTENTION

### A. The Patterson function

Inspired by some early work,^28^ we rely on deep learning solutions to directly predict the electron density map of a protein. In particular, we utilize the Patterson function,^29^ a simplified variation of the Fourier transform from structure factors to electron density, in which all structure factor amplitudes are squared, and all phases are set to zero (i.e., ignored), as in

$$p(u,v,w)=\frac{1}{V}\cdot \sum_{h,k,l}|F(h,k,l){|}^{2}\cdot e^{-2\pi i(hu+kv+lw)}.$$ (3)

It is important to note the Patterson map can be directly obtained from raw diffraction data without additional experiments or other information.

Due to the discrete size of the input and output layers in deep learning models, we can discretize and reformulate the electron density map—and its corresponding Patterson map—as follows: Suppose the electron density map of a molecule of interest is discretized into a $N_{1}\times N_{2}\times N_{3}$ 3D grid. The electron density map can then be denoted as $e\in {\mathbb{R}}^{N_{1}\times N_{2}\times N_{3}}$. The Patterson map is then formulated as follows, where ⊙ means matrix element-wise multiplication:

$$p=ℜ\left(F^{-1}\left(F(e)⊙F(\hat{e})\right)\right)\approx ℜ\left(F^{-1}\left(|F(e){|}^{2}\right)\right).$$ (4)

Breaking down the above expression, $F(e)⊙F(\hat{e})\approx |F(e){|}^{2}$ denotes only the magnitude part of the complex signals, as measured through the Fourier transform of the input signal **e**. Here, $\hat{e}$ denotes an inverse-shifted version of **e**, where its entries follow the shifted rule as in ${\hat{e}}_{i,j,k}=e_{N-i,N-j,N-k}$.

### B. Using deep learning

>We follow a data-centric approach and train a deep learning model, abstractly represented by g(θ,·), such that given a Patterson map **p** as input, it generates an estimate of an electron density map, that resembles closely the accurate map **e**. Formally, given a data distribution D and ${\left\{p_{i},e_{i}\right\}}_{i=1}^{n}∼D$, where $p_{i}\in {\mathbb{R}}^{N_{1}\times N_{2}\times N_{3}}$ is the Patterson map that corresponds to the true data electron density map, $e_{i}\in {\mathbb{R}}^{N_{1}\times N_{2}\times N_{3}}$, deep learning training aims in finding $\theta^{⋆}$ as in

$$\theta^{⋆}=\underset{\theta }{\text{arg}\;\text{min}}\;\left\{L(\theta ):=\frac{1}{n}\sum_{i=1}^{n}\ell (\theta ;\;g,\{p_{i},e_{i}\})=\frac{1}{n}\sum_{i=1}^{n}||g(\theta ,p_{i})-e_{i}|{|}_{2}^{2}\right\}.$$ (5)

Since we have a regression problem, we can use mean squared error (MSE) as the loss function L(θ).

#### 1. Using partial protein structures

Due to the well-studied structure of amino acids, we aim to optionally utilize standardized *partial structures* to aid prediction when they are available. For example, let $u_{i}^{j}\in {\mathbb{R}}^{N_{1}\times N_{2}\times N_{3}}$ be the known standalone electron density map of the *j*th amino acid of the *i*th protein example, in a specific standardized conformation, i.e., the most common rotamer of the residue as obtained from the “Get Monomer” feature of the Coot program.^30^ The number of *partial structures* associated with a protein example is equal to the number of amino acid residues present in the example, during both training and inference. Abstractly, we then aim to optimize

$$\theta^{⋆}=\underset{\theta }{\text{arg}\;\text{min}}\;\left\{L(\theta ):=\frac{1}{n}\sum_{i=1}^{n}\ell (\theta ;\;g,\{p_{i},e_{i},u_{i}^{j}\})=\frac{1}{n}\sum_{i=1}^{n}||g(\theta ,p_{i},u_{i}^{j})-e_{i}|{|}_{2}^{2}\right\}.$$ (6)

#### 2. Challenges and design principles

We face the difficult learning problem of inferring electron density maps **e** from Patterson maps **p**, which involves Fourier transformations. *These transformations can be intuitively considered as transforming local information to global information*, which is rare in common deep model use cases. Second, it is nontrivial to incorporate the partial structure density maps $u_{i}^{j}$ to aid prediction. Third, the 3D data format of both our inputs and outputs often substantially increases the computational requirements. Finally, since part of our contributions is creating novel datasets for this problem, we need to be data efficient due to the expensive dataset creation cost. Thus, the main design principles for our model can be summarized as

• *Design Principle #1*: Be able to process the global information in Patterson maps to infer the corresponding electron density maps correctly;
• *Design Principle #2*: Be able to incorporate partial structure information, when available;
• *Design Principle #3*: Learn to fulfill the above with minimal computational and data storage costs.
• *Design Principle #4*: Use existing crystallographic programs to perform refinement starting from our inferred electron density maps, obtaining a set of atomic coordinate positions.

#### 3. Gaps in current knowledge

As an initial attempt, the well-established convolution-based U-Net model^31^ could be utilized for this task. This is the path followed in our previously published work.^10^ However, classical U-Nets cannot fulfill the design principles above since: (i) they mostly rely on local information within CNN layers; such a setup is unsuitable when Patterson maps are available since the latter do not have meaningful local structures. (ii) It is not clear (or, at best, nontrivial) to incorporate any partial protein structures prior information since the latter is in a different representation domain, compared to Patterson maps. Finally, (iii) a large 3D U-Net model is computationally expensive and inefficient due to the 3D filter convolution computation.

### C. Our proposal: CrysFormer

We propose CrysFormer, a novel, 3D Transformer model^32,33^ with a new self-attention mechanism to process Patterson maps and partial protein structures to infer electron density maps with reduced costs directly, implemented in the PyTorch deep learning framework. Inspired by recent research on the potential connection between Fourier transforms and the self-attention mechanism found in the Transformer models,^34^ CrysFormer captures the global information in Patterson maps and “translates” it into correct electron density map predictions, via our proposed self-attention mechanism (*Design Principle #1*). CrysFormer does not need an encoder–decoder structure^32^ and artificial information bottlenecks^35^—as in the U-Net architecture—to force the learning of global information. By definition, CrysFormer can handle additional partial structure information that comes from a different domain than the Patterson maps (*Design Principle #2*; more details below). We can significantly reduce the overall computation cost using efficient self-attention between 3D image patches. Detaching our model from an encoder–decoder architecture further reduces the required depth of the model and, thus, the overall training cost (*Design Principle #3*). Finally, the electron density predictions produced by CrysFormer can be used as input into various crystallographic refinement programs to generate estimates of actual atomic coordinate positions (*Design Principle #4*).

### D. The architecture of the CrysFormer

**FIG. 1.**
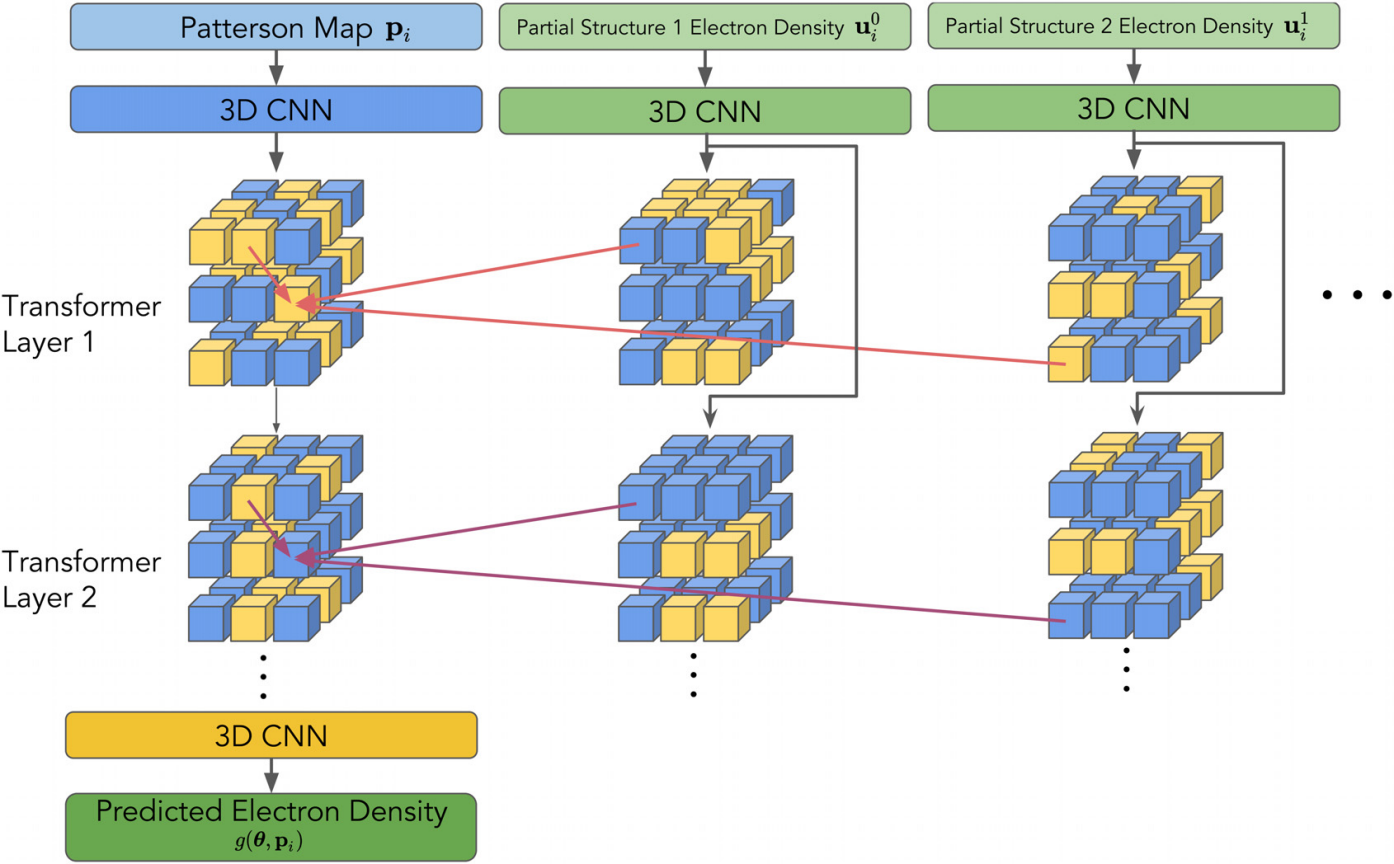
Abstract depiction of the Crysformer, which utilizes a one-way attention mechanism (red and purple arrows) to incorporate partial structure information. The tokens from the additional partial structures come from an initial 3D CNN embedding and are not passed to the next layer.

The logic for our choice of a hybrid Convolutional neural network (CNN) and Visual Transformer architecture stems from prior work in spatial image processing^33^ (but for our work, we use three-dimensional constructs instead of 2D images). We pass whole 3D Patterson map inputs $p_{i}\in {\mathbb{R}}^{1\times N_{1}\times N_{2}\times N_{3}}$ through a 3D CNN that preserves the height, width, and depth input dimensions while expanding the number of channels. As per standard practice, we then partition the CNN output into a set of smaller patches, flatten these patches into one-dimensional “word tokens,” and feed them into a multi-layer, encoder-only Transformer module. If partial structures $u_{i}^{j}$ are also available, then since they belong to a separate space from the Patterson map inputs, we send them through a separate set of convolutional and patch embedding layers, producing additional tokens that are sent to each self-attention layer. This way, the tokens in each layer can also “attend” the electron density of partial structures as a reference for final global electron density map predictions. Finally, we utilize another set of 3D convolutional layers to transform rearranged word tokens back into a 3D electron density map (see Fig. 1).

Mathematically, we report the following: The first part is the preprocessing and partitioning of input Patterson maps **p** and additional partial structures $u^{j}$ into patches of size $c\times d_{1}\times d_{2}\times d_{3}$, where *c* denotes the number of output channels of the initial preprocessing CNNs and $d_{1},d_{2},d_{3}$ denote the height, width, and depth dimensions of the patches, respectively. We flatten those patches into one-dimensional token tensors and then use a small multi-layer perceptron (MLP; a network for which all layers with trainable parameters are fully connected) to change the dimensionality of these tokens to dimension *d_t_*, where *d_t_* denotes the length of the word tokens passed to the transformer. We also add them with a learned positional embedding; this holds for both those derived from Patterson maps and partial structures, as shown below. Also, in practice, our partitioning and flattening operations can be performed together in a single tensor reordering layer for time and space savings. The operations that produce our tokens depend on the orientation and position of the features within the post-convolution maps, and are not invariant to rigid-body transformations thereof.

**Table:** 

Patterson maps p	Partial structures $u^{j}$
$\begin{array}{l} X^{0}=\mathrm{3DCN}N_{W_{c}}(p)\in {\mathbb{R}}^{c\times N_{1}\times N_{2}\times N_{3}}X^{0}=\mathrm{Partition}(X^{0})\in {\mathbb{R}}^{\frac{N_{1}N_{2}N_{3}}{d_{1}d_{2}d_{3}}\times c\times d_{1}\times d_{2}\times d_{3}}X^{0}=\mathrm{Flatten}(X^{0})\in {\mathbb{R}}^{\frac{N_{1}N_{2}N_{3}}{d_{1}d_{2}d_{3}}\times (cd_{1}d_{2}d_{3})}X^{0}=\mathrm{ML}P_{W_{c}}(X^{0})\in {\mathbb{R}}^{\frac{N_{1}N_{2}N_{3}}{d_{1}d_{2}d_{3}}\times d_{t}}X^{0}=X^{0}+\mathrm{PosEmbedding}(\frac{N_{1}N_{2}N_{3}}{d_{1}d_{2}d_{3}}) \end{array}$	$\begin{array}{lllll} U^{j}=\mathrm{3DCN}N_{W_{p}}(u^{j})\in {\mathbb{R}}^{c\times N_{1}\times N_{2}\times N_{3}}U^{j} & =\mathrm{Partition}(U^{j})\in {\mathbb{R}}^{\frac{N_{1}N_{2}N_{3}}{d_{1}d_{2}d_{3}}\times c\times d_{1}\times d_{2}\times d_{3}}U^{j} & =\mathrm{Flatten}(U^{j})\in {\mathbb{R}}^{\frac{N_{1}N_{2}N_{3}}{d_{1}d_{2}d_{3}}\times (cd_{1}d_{2}d_{3})}U^{j} & =\mathrm{ML}P_{W_{p}}(U^{j})\in {\mathbb{R}}^{\frac{N_{1}N_{2}N_{3}}{d_{1}d_{2}d_{3}}\times d_{t}}U^{j} & =U^{j}+\mathrm{PosEmbedding}(\frac{N_{1}N_{2}N_{3}}{d_{1}d_{2}d_{3}}) \end{array}$

As shown in Fig. 1, we design an efficient attention mechanism such that (i) only tokens from Patterson maps attend tokens from the partial structures; (ii) the tokens from the additional partial structures are not passed to the next layer. This is because the model should use the partial structure electron density information as a stable reference to attend to in each layer.

This one-way attention also greatly reduces the overall communication cost. In particular, let the token sequence length be $S=\frac{N_{1}N_{2}N_{3}}{d_{1}d_{2}d_{3}}$ and let *d_h_* denote the dimension of the attention head. Assuming we have *H* attention heads and *L* layers, CrysFormer uses the following attention mechanism:

$$\begin{array}{c} U=\mathrm{Conca}t_{j=1}^{J}(U^{j})\in {\mathbb{R}}^{(SJ)\times d_{t}}\\ A^{h}=\mathrm{Softmax}\left({\left(W_{q}^{h}X^{\ell }\right)}^{⊤}(\mathrm{Concat}(W_{k}^{h}X^{\ell },W_{k^{\prime }}^{h}U)\right)\in {\mathbb{R}}^{S\times (S+SJ)};\\ {\hat{V}}^{h}=A^{h}\left(\mathrm{Concat}(W_{v}^{h}X^{\ell },W_{v\prime }^{h}U)\right)\in {\mathbb{R}}^{S\times d_{h}};\\ O=W_{o}\mathrm{Concat}\left({\hat{V}}^{0},\;{\hat{V}}^{1},\ldots ,\;{\hat{V}}^{H-1}\right)\in {\mathbb{R}}^{S\times d_{t}};\\ X^{\ell +1}=W_{\mathrm{ff}2}(\mathrm{ReLU}(W_{\mathrm{ff}1}O)), \end{array}$$

where, omitting the layer index, $W_{q}^{h},\;W_{k}^{h},\;W_{v}^{h}$ are the trainable query, key, and value projection matrices of the *h*th attention head for tokens from the Patterson map, and $W_{k^{\prime }}^{h},\;W_{v^{\prime }}^{h}$ are the corresponding matrices for tokens from the partial structure, each with dimension *d_h_*. Furthermore, $W_{\mathrm{ff}1}$ and $W_{\mathrm{ff}2}$ are the trainable parameters of the fully connected layers. We omit skip connections and layer normalization modules to simplify notation, but these are included in practice.

For a final step, we transform the output embeddings of our final transformer layer back to a 3D electron density map, as follows:

$$g(\theta ,p)=\tanh(\mathrm{3DCN}N_{W_{o}}(\mathrm{Rearrange}(\mathrm{MLP}(X^{L}))))\in {\mathbb{R}}^{N_{1}\times N_{2}\times N_{3}}.$$

As our loss function L(θ) between model outputs g(θ,p) and ground truth electron density maps **e**, we use a combination of the previously mentioned mean squared error and the negative Pearson correlation coefficient. The Pearson correlation is a widely used metric in crystallography. If we denote a model prediction as e′ and define $\bar{e}=\frac{1}{N_{1}N_{2}N_{3}}\sum_{i,j,k}e_{i,j,k}$ and $\bar{e}\prime =\frac{1}{N_{1}N_{2}N_{3}}\sum_{i,j,k}e\prime_{i,j,k}$, then the Pearson correlation coefficient between **e** and e′ is as follows:

$$\mathrm{PC}(e,e\prime )=\frac{\sum_{i,j,k=1}^{N_{1},N_{2},N_{3}}\left(e\prime_{i,j,k}-\bar{e}\prime )(e_{i,j,k}-\bar{e}\right)}{\sqrt{\sum_{i,j,k=1}^{N_{1},N_{2},N_{3}}\left[{\left(e\prime_{i,j,k}-\bar{e}\prime \right)}^{2}\right]+\epsilon }\cdot \;\sqrt{\sum_{i,j,k=1}^{N_{1},N_{2},N_{3}}[{\left(e_{i,j,k}-\bar{e}\right)}^{2}]+\epsilon }},$$ (7)

where *ϵ* is a small constant to prevent division by zero. Larger values of the Pearson correlation correspond to more accurately predicted electron density maps. Thus, to incorporate it as a term in our loss function, we use its sign negation. Our overall loss is heavily weighted in favor of the MSE, as we weigh the MSE by 0.9999 and the negative Pearson correlation by $1\times 10^{-4}$.

### E. Completion: Map interpretation and generation of atomic coordinates

The last step in the pipeline is to determine atomic coordinates for the maps and determine if they can be refined using traditional methods. Powerful tools exist for interpreting reasonably well-phased electron density maps. The last step in our model interprets the maps and generates an appropriate set of atomic coordinates. We use either the *Autobuild* feature within the *PHENIX* suite^36,37^ or the poly-alanine autotracing option in *SHELXE.*^38,39^

The procedure using *PHENIX* is quite computationally expensive, so we could only perform the process on a randomly selected subset of the test set. We first performed fitting while holding the map to be fitted constant instead of iterative phase improvement to avoid bias from the direct power of solvent flattening and histogram matching. For the simple fitting analysis, the default *AutoBuild* parameters were used with the exception that the NCS flag (non-crystallographic symmetry) was set to FALSE and the *USE_CONSTANT_INPUT_MAP* flag was set to TRUE. These jobs then performed three build cycles on each test example, including loop fitting, connecting segments, building outside the initial models, and searching for secondary structures. The overall best set of coordinates was saved based on the R-free value during the process (see Sec. V D).

In another trial on members of the test set, the default *AutoBuild* was used with no restrictions on density modification. In this trial, the model was iteratively refit using improved phases. In a third trial, *PHENIX* map interpretation was performed starting from the best coordinate sets in the initial fixed-map step. The flag *TWO_FOFC_IN_REBUILD* flag was set to TRUE so that the structure could be refined from the initial unbiased interpretation but evolve the phases based only on the model and its refinement, see also in Sec. V D.

For the *SHELXE* method, which is considerably less computationally expensive on a per-case basis, we evaluated all 16 230 members of the test set. This method uses density modification to improve the phases during fitting and results in poly-alanine backbone models from which diffraction amplitudes can be calculated and compared to the original “experimental” amplitudes from the test set. For each test case, eight global tracing cycles with ten cycles of density modification were run during each global cycle. Density modification involved negative density truncation in the protein region and density flipping in the solvent with a weighted combination for voxels with intermediate scores for protein vs solvent identified using the sphere-of-influence method.^38^

## IV. NEW DATASETS

We collect coordinate sets for hypothetical short protein fragments derived from Protein Databank (PDB) entries,^7^ then generate corresponding input Patterson and output electron density maps using an appropriate unit cell. We start from a curated basis of ∼24 000 such protein structures. Then, we randomly select and store segments of adjacent amino acid residues from a random subset of about half of these structures. These examples comprise dipeptides (two residues) and 15-mers, leading to two datasets we introduce with this work. We start with two residues for a more straightforward initial problem, as in previous work,^10^ and increase to 15 residues as an intermediate step toward realistic problems. For the latter dataset, three residues could be shared between different examples. Using the pdbfixer Python API,^40^ we remove all examples containing nonstandard residues or missing atoms from our initial set.

For our dipeptide dataset, we then iteratively expand the unit cell dimensions for each example, starting from the raw max−min ranges of Cartesian coordinates in each of the three axis directions, attempting to create a minimal-size unit cell where the minimum intermolecular atomic contact is at least 2.75 Angstroms (Å). After this, all examples containing clashes with atomic contacts of less than 2.75 Å are discarded. For our 15-residue dataset, we instead place atoms in fixed unit cells of size 41 Å × 30 Å × 24 Å to simplify the now much harder problem; this exact cell size was chosen such that the vast majority ( ∼97%) of examples would satisfy our 2.75 Å atomic contact threshold. Since these unit cells are relatively large compared with the corresponding protein fragments, solvent content is unusually high ( >90%), so our examples admittedly can be readily solved with aforementioned direct methods. The examples are then reoriented via a reindexing operation, such that the first axis is always the longest and the third axis is always the shortest.

If ambiguous inputs are provided to an ML algorithm, then training often proceeds poorly or not at all, and one issue leading to potential ambiguity in interpreting Patterson maps is their invariance to translation of the entire corresponding electron density.^28^ To tackle this, we center all atomic coordinates such that the center of mass is in the center of the corresponding unit cell, and so all of our structures are generally located in the middle of the cell, with empty gaps closer toward the cell boundaries. Thus, our model also learns to predict electron densities that are always more or less centered in the unit cell. This does allow us to directly calculate Pearson correlations during evaluation and as a loss function term, without needing to first align origins.

Structure factors for each remaining example, as well as those for the corresponding partial structures for each of the present amino acids, are generated using the gemmi sfcalc program^41^ to a resolution of 1.5 Å. An electron density and Patterson map for each example are then obtained from those structure factors with the fft program of the CCP4 program suite;^42,43^ partial structure densities are obtained in the same manner. We specify a grid oversampling factor of 3.0, resulting in a 0.5 Å grid spacing in the produced maps. All these maps are then converted into PyTorch tensors. We then normalize the values in each tensor to be in the range [−1, 1]. Since, in our PyTorch implementation, all examples within a training batch are the same size, we remove all examples from the tensor-size bins containing fewer examples than a specified minimum batch size.

We have created a repository of our training and dataset generation scripts, as well as intermediate atomic coordinate files corresponding to our 15-mer dataset. These scripts and coordinate files can be used to produce our 15-mer training and test sets, as described below. This repository can be found at https://github.com/sciadopitys/CrysFormer.

## V. EXPERIMENTS

### A. Baselines

There are no readily available off-the-shelf solutions for our baseline setting, as our work is novel. As our baseline for comparison, we use our previous CNN-based U-Net model;^10^ this architecture is widely used in image transformation tasks.^31,44^

For comparison, we have further enhanced this vanilla U-Net with (i) additional input channels to incorporate the partial structure information, despite being potentially unsound; and (ii) a “recycling” model procedure, which retrains a U-Net re-initialization using previous model predictions as additional input channels alongside the Patterson maps. Both of these extensions significantly improve the performance of the vanilla U-Net. We refer the reader to the Appendixes for more details on our baseline model architecture.

### B. Metrics

During evaluation, we calculate the Pearson correlation coefficient, as defined previously, between ground truth targets **e** and model predictions g(θ,p). To further demonstrate how well our methods solve the phase problem, we perform phase error analysis on our models' final post-training predictions using the cphasematch program of the CCP4 program suite.^45^ We report the mean phase errors of our predictions in degrees, as reported by cphasematch, where a smaller phase error is desirable. Finally, we compare the convergence speed and computation cost of both methods.

### C. Results on dimer peptide models

A summary of our results on our dipeptide dataset, which consisted of 1 894 984 training and 210 487 test cases, is provided in Table I. Overall, CrysFormer significantly improves prediction accuracy regarding the Pearson coefficient and mean phase error while requiring a shorter time (in epochs) to converge. CrysFormer also incurs much less computation cost, significantly reducing wall clock time per epoch. However, CrysFormer does incur a slightly higher maximum memory usage than U-Net.

**TABLE I.** CrysFormer vs baselines on the dipeptide dataset. U-Net+R refers to adding the recycling procedure to U-Net training; U-Net+PS+R refers to adding further partial structures as additional channels. Boldface type represents our best model to date.

Method	Mean PC(e,e′)	Mean phase error	Epochs	Time per epoch (min)
U-Net ^10^	0.735	67.40°	50	28.93
U-Net+R (This work)	0.775	58.67°	90	29.06
U-Net+PS+R (This work)	0.839	51.34°	90	29.31
CrysFormer (This work)	**0.939**	**35.16**°	**35**	**12.37**

**FIG. 2.**
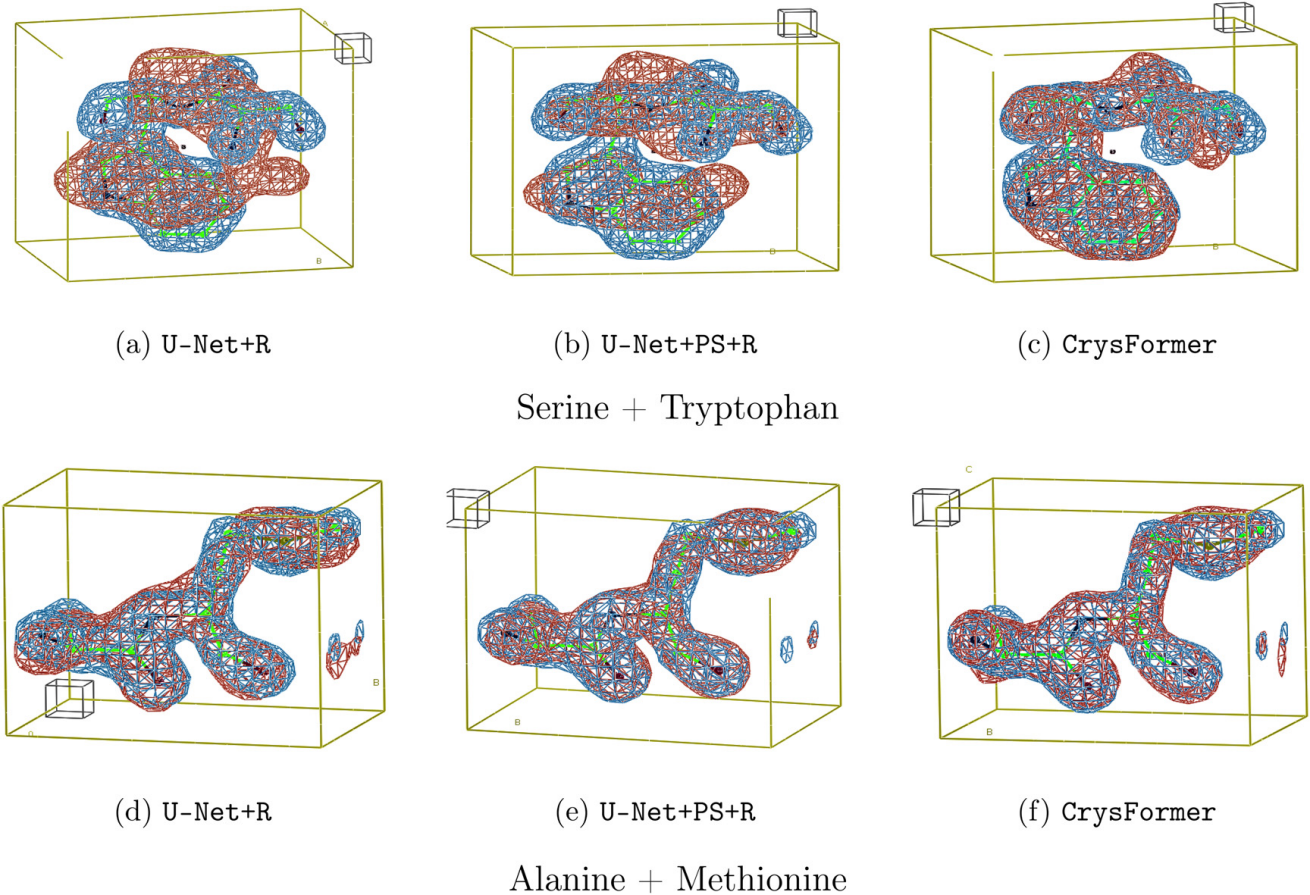
Example visualizations of electron density predictions for earlier baseline models and CrysFormer: Ground truth density maps are shown in blue, while predictions are shown in red. The model used to generate the ground truth electron density is shown in stick representation for reference. (a) and (d) The convolution U-net plus a retraining run, (b) and (e) the convolution U-net plus an additional channel with partial structures and retraining, (c) and (f) the CrysFormer attention model.

We further visualize some predictions in Fig. 2, comparing those made by the baselines and the CrysFormer. CrysFormer produces more accurate predictions in terms of both global and local structures. This verifies our hypothesis that (i) the self-attention mechanism can better capture the global information in Patterson maps, and (ii) the removal of the U-Net's encoder–decoder structure prevents loss of information and improves the reproduction of finer details.

For example, the top row of Fig. 2 represents a class of examples containing a large aromatic residue, Tryptophan. U-Net+R models consistently produce poor predictions in this case, while the CrysFormer better handles such residues. U-Net+PS+R shows that both providing additional input channels and using the retraining procedure improves results even for U-Net architectures; yet, CrysFormer still provides better reconstruction. Many visualizations can be found in the Appendixes.

**FIG. 3.**
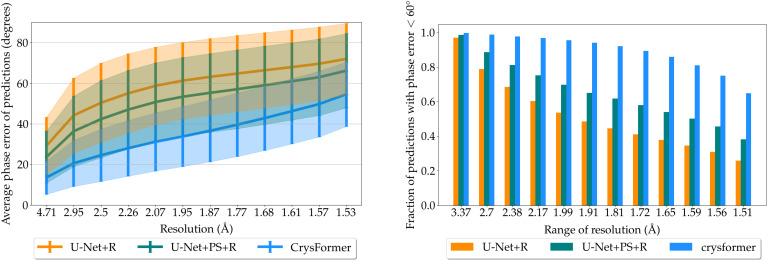
Dipeptide dataset analysis. Left: Average phase error of model predictions from the test set plotted vs diffraction resolution. Right: Fraction of test model predictions for which phase error is <60° at various ranges of resolution. The results show well-phased structures to the limit of resolution of the test samples for the attention-based CrysFormer method.

We further plot the calculated average mean phase errors of the predictions of our models against reflection resolution, see left panel of Fig. 3. The predictions by CrysFormer have lower mean phase error than baselines. This means that, on average, the CrysFormer predictions can better reproduce the general shape and finer details of the ground truth electron densities.

Finally, we generate a chart of the fraction of our models' predictions for which the calculated mean phase error is <60° at various resolution ranges. We consider such predictions to accurately reproduce the level of detail specified by that resolution range. Figure 3 shows this on the right panel. At all resolution ranges, CrysFormer predictions are better than those of the U-Net-based models. In particular, for CrysFormer, we still have a majority of predictions with phase error <60° even at the highest ranges of resolution.

### D. Results on the 15-mer set of structures

On our dataset of 15-residue examples, which consisted of only 165 858 training and 16 230 test cases (less than one-tenth the size of our dipeptide dataset), we trained for 80 epochs to a final average test set Pearson correlation of about 0.747. We then performed a recycling training run of 20 epochs, incorporating the original training run's predictions as additional input channels, again alongside the Patterson maps, when training another CrysFormer, and obtained an improved average test set Pearson correlation of about 0.77 and phase error of about 67.66. On both of these runs, we made use of the PyTorch implementation of the Nyström approximate attention mechanism,^46^ slightly modified to work with our partial structure inputs, to reduce time and space costs. Each training epoch still took about 6.28 h to complete. Thus, due to time considerations, we decided not to attempt to train a U-Net on this dataset for comparison purpose.

**FIG. 4.**
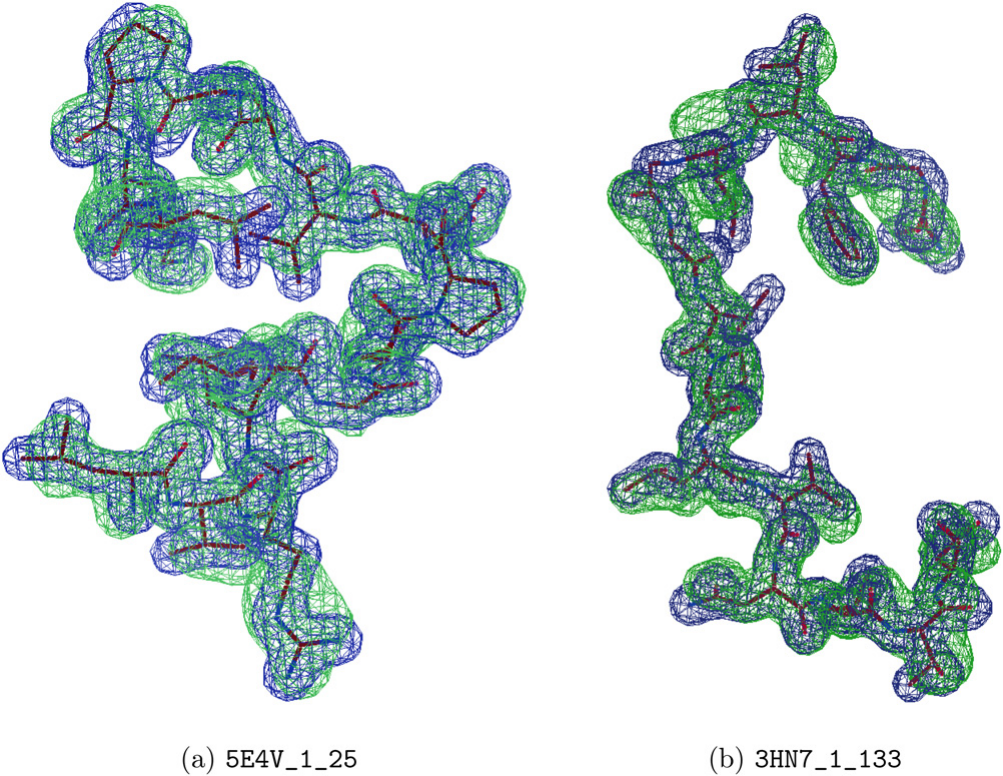
Example visualizations of two successful test predictions after the CrysFormer retraining was performed; (a) with Pearson correlation 0.90 and percentile rank 82% and (b) with correlation 0.83 and percentile rank 55%. Ground truth density maps shown in blue and predictions shown in green. The model used to generate the ground truth electron density map is again shown in stick representation in black for reference.

**FIG. 5.**
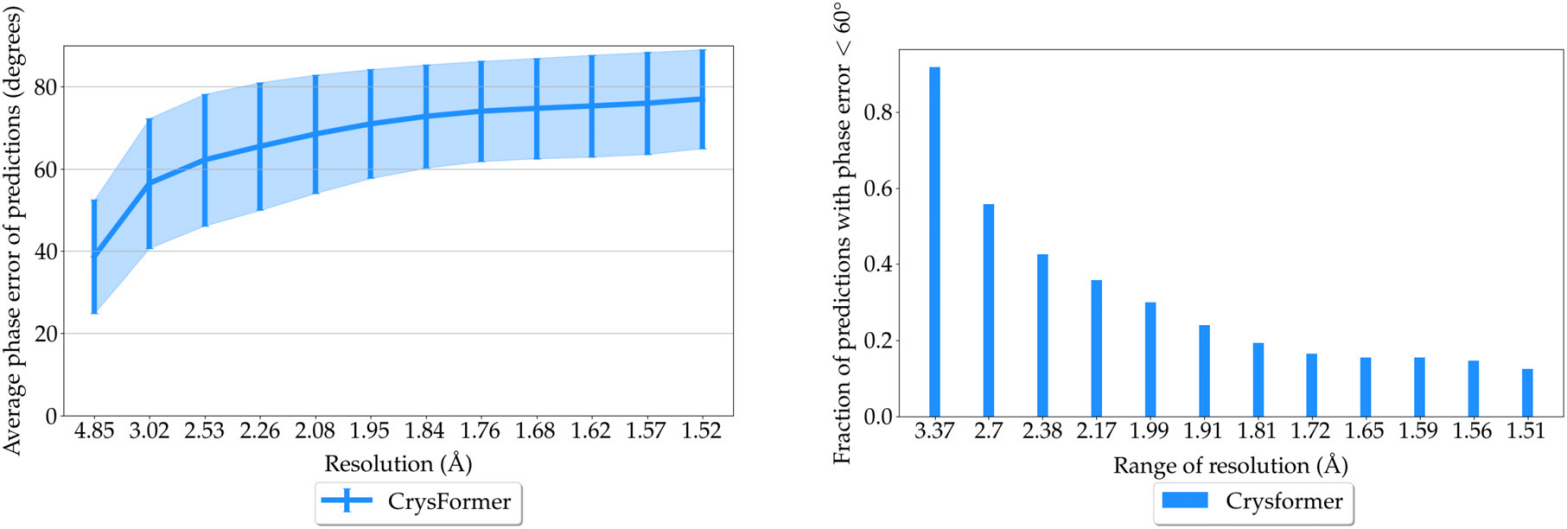
Analysis of the phase errors for the CrysFormer model on a dataset of 15-mer peptide models. Left: Average phase error of model predictions vs diffraction reflection resolution. Right: Fraction of model predictions on a 15-residue dataset for which phase error is <60° at various ranges of resolution. The data suggest that a large fraction of structures were well-phased to a resolution of 2.7 A or better, and a smaller fraction was well-phased to the resolution limit.

We provide visualizations of some model predictions in Fig. 4; more can be found in the Appendixes. We also plot the average mean phase errors of the predictions of our models against reflection resolution, as well as the fraction of our models' predictions for which the calculated mean phase error is <60° at various ranges of resolution in Fig. 5. These results show that this is a more challenging dataset with reduced sample size, yet CrysFormer predictions tend to reproduce most details of the desired electron densities accurately.

**FIG. 6.**
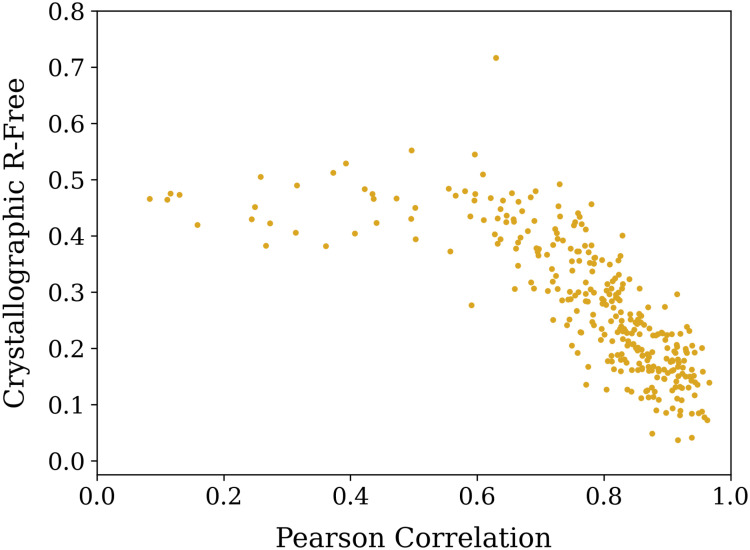
Analysis of R-free using an atomic model fitted to a fixed input map vs Pearson correlation coefficient between the ground truth map and the predicted map for a subset of the test cases. The scatterplot shows a large fraction of values in the lower right quadrant, demonstrating a strong correlation between the directly predicted map and the ability to interpret the map as accurate atomic coordinates.

**FIG. 7.**
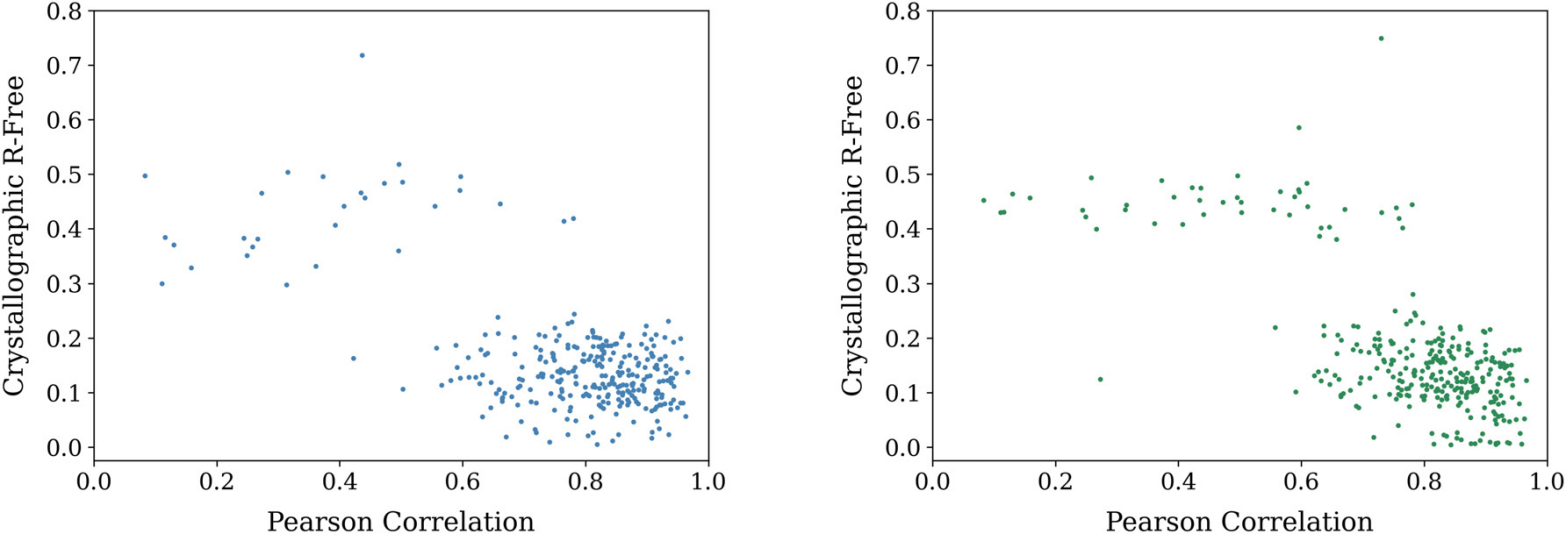
The general success of the CrysFormer is demonstrated by successfully interpreting the predicted electron density maps of the test set to produce atomic structures. The movement of points downward relative to the raw results shown in Fig. 6 proves the structures have been solved in the crystallographic sense. Left: Scatterplot of post-refinement R-free values vs correlation between the predicted and ground truth maps, after full-scale *AutoBuild* refinement was applied. Right: Scatterplot of post-refinement R-free vs map correlation values, starting with the coordinates derived from only a CrysFormer-supplied fixed input map.

We used the *Autobuild* program within the *PHENIX* suite^36,37^ to perform automated interpretation of a randomly selected set of 302 maps from the test set after the recycling training run. Even if no iterative rebuilding was performed, and instead atomic models were fitted only to the fixed predicted electron density maps directly from the CrysFormer, we found that 229 out of 302 ( ∼76%) could refine to a crystallographic *R*-free better than 0.38, as shown in Fig. 6. Fully automated model building and crystallographic refinement, including density modification, did better, where we found that 281 out of 302 ( ∼93%) refined to a final atomic model with a crystallographic *R*-free of less than 0.38. When starting from the best models from the fit only to the fixed map and using only 2FoFc map evolution and refitting and no interactive density modification, 258 out of 302 ( ∼85%) refined to such an *R*-free. Figure 7 shows these results as scatterplots; clearly, only a tiny fraction of the subset of predictions did not refine successfully, even when solvent flattening and density modification were not used in the early phases of map interpretation.

**FIG. 10.**
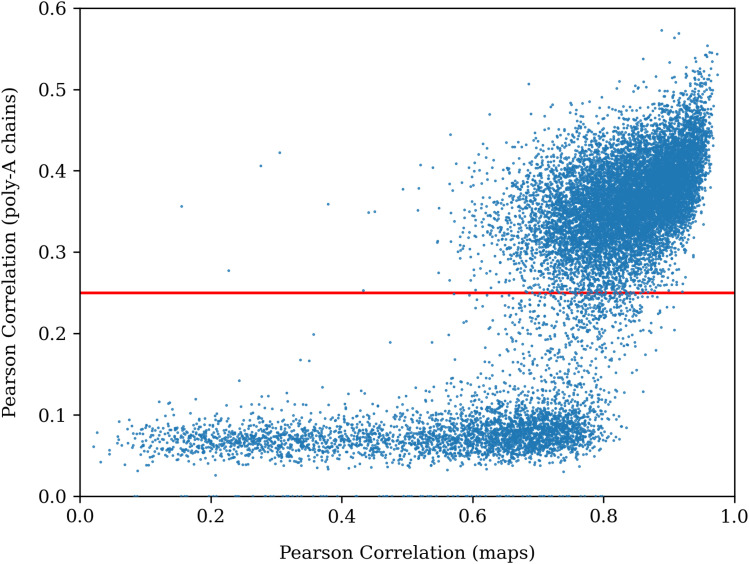
Scatterplot of the Pearson correlations of ground truth vs model-based diffraction amplitudes for the poly-alanine chains auto traced by *shelxe* for all 16 230 test cases. The results show that most cases (upper right cloud) were “solved” correctly by the Crysformer as they could be reduced to coordinates whose calculated diffraction matches the ground truth diffraction pattern.

Furthermore, after automatic map interpretation using the auto building routines in *shelxe*^38^ to obtain a poly-alanine chain from ALL of the 16 230 test set predictions, we found that almost 74% of the resulting models had calculated diffraction amplitudes with a Pearson correlation coefficient of at least 0.25 to the true underlying data (see Fig. 10). While not quite as good as the *PHENIX* interpretations, historical results indicate that further refinement from the poly-alanine model would produce a “correct” model.

## VI. DISCUSSION

We have shown that CrysFormer outperforms state-of-the-art models for predicting electron density maps from corresponding Patterson maps in all metrics on a newly introduced dataset (dipeptide). Overall, CrysFormer requires fewer epochs to converge reasonably and has a smaller computational footprint. Furthermore, our “recycling” procedure significantly improves training for the vanilla U-Net architecture on our dipeptide dataset, as well as for training CrysFormer on both our dipeptide and 15-residue datasets.

### A. Limitations and next steps

While our method is not yet useful in providing practical real-world use over existing methods, our successful results on our initial 15-residue dataset are an intermediate step toward our goal of solving actual proteins given crystallographic data. To build upon this, we must progressively make our dataset examples closer to true protein crystal structures. To this end, we suggest training our model on examples with variable unit cells as future work. Afterward, we aim to handle variable cell angles and then train on examples with different types of internal symmetry, i.e., belonging to space groups other than P1. Yet, another form of variability we will introduce is allowing the grid spacings and resolution limits of our examples to vary. Finally, we will explore changing the formulation of our partial structures to have more than one amino acid residue in a structure, as having each partial structure represent only a single residue may no longer be reasonable, both computationally and from a practical perspective, as we move to more complex datasets.

### B. Broader impacts

Solving the crystallographic phase problem for proteins would dramatically reduce the time and expense of determining a new protein structure, especially if no close homologs are already in the Protein Data Bank. Some methods sometimes work under particular conditions,^47^ and sometimes work but only at very low resolutions.^48^ The recent line of work on AlphaFold^8,49^ helps in these problems, especially in cases where reliable predictions are possible due to strong homologs and extensive sequence data, or when predictions themselves are used as input into established crystallographic methods such as molecular replacement and refinement.

## Figures and Tables

**FIG. 8.**
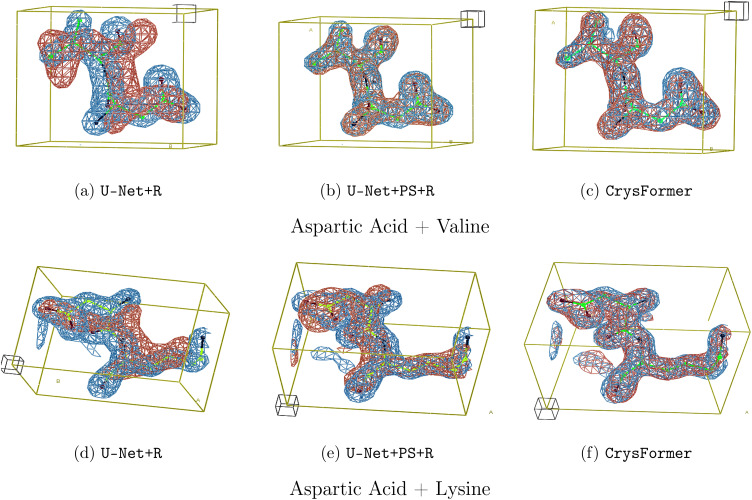
More test visualizations for the dipeptide dataset. (a) and (d) The convolution U-net plus a retraining run, (b) and (e) the convolution U-net plus an additional channel with partial structures and retraining, (c) and (f) the CrysFormer attention model.

**FIG. 9.**
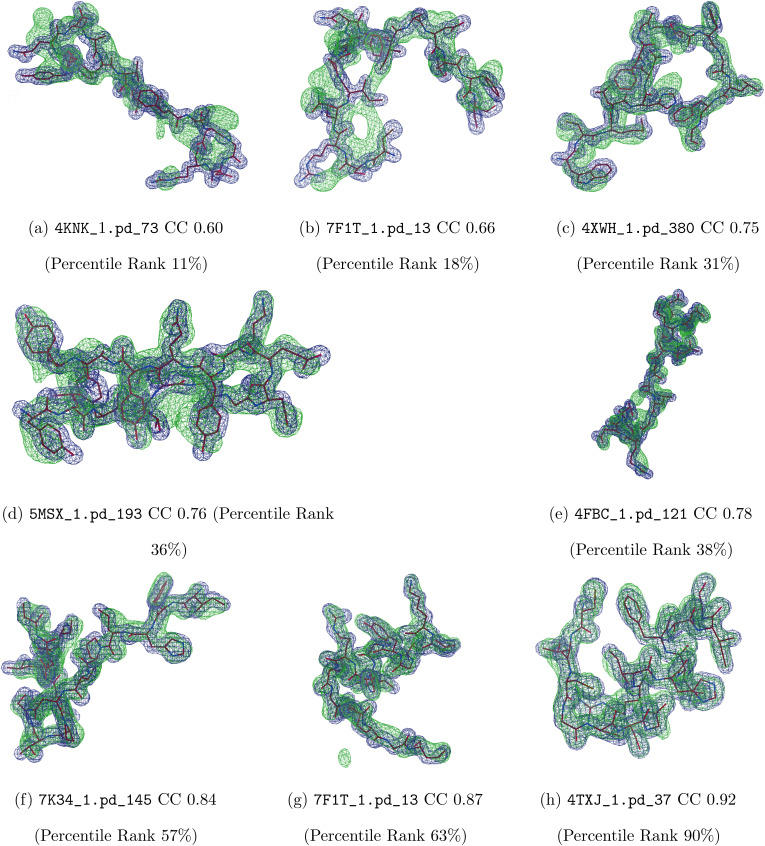
More test prediction visualizations for the 15-residue dataset. See sublabels for Pearson correlation and percentile rank of examples.

## Data Availability

The data that support the findings of this study are openly available in GitHub at https://github.com/sciadopitys/CrysFormer, (Ref. 53).

## APPENDIX A: MODEL ARCHITECTURE OF BASELINE U-NET MODEL

Our U-Net architecture can be divided into three phases. The *Encoding Phase* consists of two 7 × 7 × 7 convolutional layers. The first has 25 output channels, while the second has 30 output channels, and both are followed by the standard batch normalization and a ReLU activation. Then, a max pooling operation with kernel size 2 × 2 × 2 and stride two is used to reduce the height, width, and depth dimensions by 2.

The *Learning Features Phase* consists of a sequence of seven residual blocks. Each of these blocks consists of a 7 × 7 × 7 convolutional layer with 30 output channels followed by batch normalization and ReLU activation, and then another 30-channel 7 × 7 × 7 convolutional layer with batch normalization but no activation. A squeeze and excitation block^50^ occurs at this point, applied with the channel dimension reduced by a factor of 2. Afterward, the residual skip connection is applied, followed by another ReLU activation. At the end of this phase, a naive upsampling operation is used to increase the height, width, and depth dimensions by a factor of 2, thus restoring the original dimensions.

The *Decoding Phase* consists of two 5 × 5 × 5 convolutional layers. The first has 25 output channels and is followed by batch normalization and a ReLU activation, while the second produces the model predictions and thus has only a single output channel. Since all elements of the target outputs were constrained to be in the range [−1], we apply a final tanh activation function after this layer.

In all convolutional layers, the input is “same” padded to preserve height, width, and depth dimensionality. Also, the convolutional layers in the encoding and learning features phases are padded using PyTorch's circular padding scheme to account for the periodic nature of the input Patterson maps. Furthermore, all convolutional layers were initialized using the kaiming_normal function of the default torch.nn module.^51^ As with the CrysFormer, our U-Net model is robust to training batches of examples with differing height, width, and depth shapes.

## APPENDIX B: ADDITIONAL DETAILS ON DATASET GENERATION

To start preparing our dataset, we selected nearly 24 000 representative Protein Data Bank (PDB) entries using the following criteria: proteins solved by x-ray crystallography after 1995, sequence length ≥ 40, refinement resolution ≤ 2.75, refinement R-Free ≤ 0.28, with clustering at 30% sequence identity and available in legacy PDB format. We applied standardized modifications to each viable coordinate file: all temperature factors were set to 20, selenomethionine residues were rebuilt as methionine, and all hydrogen atoms were removed, leaving only carbon, nitrogen, oxygen, and potentially sulfur.

In our dataset generation process, an effort was made to ensure diversity by sampling from PDB entities with low sequence similarity to each other. However, test and training sets take random samples from the conformations allowed in the rotamer and Ramachandran space. Any similar conformations would be expected to be in a different rotational orientation in the cell by the nature of the selection process. We did not compute all-vs-all clustering or force the test and training sets to sample distinct conformational regions. For our 15-residue dataset, we allowed at most three residues to be shared between particular examples to obtain more starting coordinate files. To prevent potential overfitting from this sharing of subsegments, we enforced that all examples derived from the same initial.pdb file would be placed together in either the training or test set.

Another issue regarding ambiguity in Patterson map interpretation is that an electron density will always have the same Patterson map as its corresponding centrosymmetry-related electron density. Hurwitz^28^ provided a workaround that involved combining a set of atoms with the set of its centrosymmetry-related atoms into a single example output. However, this also requires a separate post-processing algorithm to separate the original and centrosymmetric densities for each of his model's predictions. Since we are working with real-world structures—rather than randomly placed data—we can exploit their known properties. In particular, we know that all proteinogenic amino acids are naturally found in only one possible enantiomeric configuration.^52^ Although the mirror-image symmetry of enantiomers is not the same as centrosymmetry, we show that this is enough to allow us to work with true electron densities of protein fragments.

## APPENDIX C: NOMENCLATURE OF OUR EXAMPLE IDS

Our example IDs contain three fields separated by underscore characters. The first field refers to the PDBid of the structure from which the example was taken. The second field refers to the distinct entity of the aforementioned PDB structure from which the example was taken. The third field refers to the index of the first amino acid residue present in the example, not counting any missing/unmodeled residues in the PDB structure from which it was taken.

## APPENDIX D: DESCRIPTION OF DATASET SUBSET

Due to limitations of online storage space, we provide a subset of our generated dataset. This subset represents a total of 200 000 dipeptide examples. As expected, patterson.tar.gz contains the generated Patterson maps, while electron_density.tar.gz contains the corresponding electron densities. Meanwhile, partial_structure.tar.gz has both partial structures for each dipeptide example in the subset.

The dataset can be downloaded through this link: https://drive.google.com/drive/folders/1X7YkxDd7yTC1RTG1z3NbdRIfKLfFtkrx?usp=share_link

As mentioned, we also provide a dataset of prepared.pdb coordinate files of 15-residue examples, to which our dataset generation process can be applied to produce Patterson map and electron density tensors.

## APPENDIX E: LIST OF FAILED CASES

The following 19 cases of the subset of our 15-mer test set examples were unable to be refined successfully (crystallographic R-free < 0.38) all three times we performed Autobuild interpretation after our recycling training run: 2AEB_1.pd_229, 2E5A_1.pd_109, 2O5H_1.pd_61, 2VA0_1.pd_73, 2Y69_7.pd_59, 3AM2_1.pd_97, 3FF7_2.pd_25, 3L9F_1.pd_85, 3LO8_1.pd_97, 3NS6_1.pd_73, 5AJO_1.pd_181, 5MSX_1.pd_193, 5NXQ_1.pd_227, 5TZ5_1.pd_217, 6FBM_1.pd_229, 6JDR_1.pd_265, 6LXU_1.pd_181, 6TIT_1.pd_349, 7MPZ_1.pd_61.

## APPENDIX F: ADDITIONAL VISUALIZATIONS AND ANALYSIS OF MODEL STRUCTURE PREDICTIONS

Figure 8 provides additional visualizations on our dipeptide dataset. As in the main text, ground truth density maps are in blue while predictions are in red, and the model used to generate the ground truth electron density is shown in stick representation for reference. The first row represents an example in which the additional partial structure input channels provided to the U-Net substantially increased prediction quality, allowing it to produce a prediction similar to that of the CrysFormer. The second row represents an example in which providing additional input channels to the U-Net and switching to CrysFormer provided noticeable improvements in prediction quality.

Figure 9 provides additional visualizations on our 15-mer dataset. As in the main text, ground truth density maps are in blue while predictions are in green, and the model used to generate the ground truth electron density is shown in stick representation. It is clear that as prediction quality increases, as indicated by the reported Pearson correlation, finer details of the true underlying structure are more likely to be accurately reproduced. The predictions in Figs. 9(e)–9(h), as well as Fig. 4(a) (rank 55%) and (b) (rank 82%), were all successfully refined using all of the mentioned autotracing and refinement procedures. However, even for relatively poor predictions such as (a) and (b), the rough overall shape can be reproduced even though several portions have apparent inaccuracies.

Additional methods were used to verify that structures were correctly solved by subjecting them to automatic interpretation using SHELXE. Figure 10 shows the scatterplot of *SHELXE* poly-alanine autotracing results on the full 15-residue test set. As mentioned, examples for which the amplitudes calculated from the initial poly-alanine chain built into the model electron density prediction have a Pearson correlation coefficient with the true underlying structure factor amplitudes of over 0.25 (shown above the red line) are highly likely to be successfully refined. This method uses density modification methods that include solvent flattening and thus uses phasing power beyond the CrysFormer capabilities. On this basis, we did not have this result in the main paper. The two clusters of data points correspond to solvable structures (upper right) and recalcitrant ones (along the bottom).
